# Antimicrobial Susceptibility Profiles of *Pasteurella multocida* Isolates from Clinical Cases of Waterfowl in Hungary between 2022 and 2023

**DOI:** 10.3390/vetsci11050194

**Published:** 2024-04-28

**Authors:** Ádám Kerek, Ábel Szabó, Ákos Jerzsele

**Affiliations:** 1 Department of Pharmacology and Toxicology, University of Veterinary Medicine, István utca 2, 1078 Budapest, Hungary; szabo.abel@student.univet.hu (Á.S.); jerzsele.akos@univet.hu (Á.J.); 2 National Laboratory of Infectious Animal Diseases, Antimicrobial Resistance, Veterinary Public Health and Food Chain Safety, University of Veterinary Medicine, 1078 Budapest, Hungary

**Keywords:** *Pasteurella multocida*, antimicrobial resistance, fluoroquinolone resistant, minimum inhibitory concentration, MIC, waterfowl, geese, ducks

## Abstract

**Simple Summary:**

In this study, the antibiotic susceptibility of *Pasteurella multocida* strains isolated from clinical cases in waterfowl was examined. *Pasteurella multocida* can cause potentially severe diseases, usually due to predisposing factors. Our investigations involved determining the minimal inhibitory concentration (MIC), which is the lowest concentration of a substance that still effectively inhibits bacterial growth. Our results indicate that a significant portion of the isolates remained susceptible to most antibiotics, with some exceptions showing resistance to enrofloxacin. This study highlights the problem of antibiotic resistance and the importance of appropriate antibiotic selection for effective disease management. The findings of this research encourage further studies on antibiotic susceptibility and optimal treatment strategies to preserve waterfowl health.

**Abstract:**

The waterfowl industry represents a narrow, yet economically significant, sector within the poultry industry. Although less prominent, the waterfowl sector is nonetheless of equal importance to any other livestock sector in terms of antimicrobial resistance and animal health issues. This study assesses the antimicrobial resistance profile of *Pasteurella multocida* bacterial strains isolated from clinical cases in Hungary’s duck and goose populations, determining the minimal inhibitory concentration (MIC) of 27 samples collected from 15 different locations. The results indicate that the isolated strains were susceptible to most antibiotics, except for notable resistance to enrofloxacin. These findings support that *Pasteurella multocida* largely retained its susceptibility. However, the observed resistance to enrofloxacin suggests overuse of fluoroquinolones, which indicates the potential need for stricter regulation of their use in the poultry industry.

## 1. Introduction

Antimicrobial resistance (AMR) currently stands as a significant global health challenge, sparking concerns worldwide. Current estimates suggest that 700,000 people die annually as a result of AMR, and this number is projected to increase, potentially causing 10 million deaths per year by 2050 [1]. AMR has now become one of the top 10 global health risks [2], evolving into a “silent pandemic” [3]. The emergence of resistance can be attributed to two main factors: the high burden of infections on the healthcare system and the inconsistent use of antibiotics [4]. Resistant bacteria and their genes, which develop and are harbored in humans, animals, or pests, can easily spread among individuals and enter the environment. Active ingredients entering the soil and then drinking water can be absorbed by wildlife, further spreading and perpetuating AMR [5]. The demand for new antibacterial agents to treat multidrug-resistant (MDR) bacterial infections has been recognized by the World Health Organization (WHO) [6]. The Global Action Plan (GAP), developed by the Tripartite AMR National Action Plans of the World Organization for Animal Health (OIE), the Food and Agriculture Organization of the United Nations (FAO), and the Pan American Health Organization (PAHO), sets objectives that represent positive steps toward the sustainable management of antibiotic resistance [7]. The waterfowl sector represents a significant economic area with substantial potential, although it is often considered less prominent than the poultry sector. However, in terms of antimicrobial resistance and animal health, it is equally important as the chicken or turkey populations. In Hungary in 2022, approximately 614,000 geese and 2,727,000 ducks were registered, representing 1.76% and 7.79% of all poultry, respectively. A significant portion of the final products is sold on the export market. According to 2021 aggregated data, 151,936 tons of duck meat and 38,477 tons of goose meat were produced in Hungary. Hungarian goose meat consumption averages 70–80 g per person per year [8]. Several pathogens, including *Pasteurella multocida*, *Escherichia coli*, and *Pseudomonas aeruginosa*, have globally emerged as key pathogens in waterfowl diseases [9].

*Pasteurella multocida* is a gram-negative zoonotic pathogen that widely occurs in numerous host species worldwide. Its rapid spread among both humans and animals, coupled with its high mortality rate, causes significant economic losses in agriculture [10]. It commonly causes pneumonia, septicemia, meningitis, and eye infections, with its pathogenicity influenced by numerous virulence factors [11]. In poultry, *Pasteurella multocida* causes fowl cholera, a severe systemic infection that results in significant economic losses to the poultry industry globally [12]. Typically presenting as asymptomatic or mild chronic sinusitis and conjunctivitis, it often progresses to pneumonia, leading to a rapid fatal outcome as a disseminated disease. Predisposing factors (stress, environmental conditions, overall health) play a crucial role in its pathogenesis [13]. Asymptomatic carriage of the pathogen on the respiratory or cloacal mucosa of birds can serve as a source of outbreaks under predisposing factors [14], and the role of wild birds, rodents, and aerosols in transmission has been described [15,16,17]. In the duck industry, the frequency of healthy flocks carrying the pathogen can be as high as 63%, with mortality rates reaching up to 50% following infection under predisposing factors, which can be significantly reduced by the application of rapid diagnostic PCR tests [18].

Although most antibiotics have retained their efficacy in the treatment of *Pasteurella multocida* infections to date, increasingly resistant strains are emerging. These may well pose a significant challenge to antibiotic efficacy in the future, especially due to the emergence and spread of multidrug-resistant strains [19]. The need for responsible antibiotic use was highlighted when the first hypervirulent and multidrug-resistant strain was reported in Peking ducks in China in 2020 [20]. Furthermore, regular susceptibility monitoring is necessary to track the evolving landscape. Therefore, we aim to conduct a national survey over a one-year period to establish the antibiotic susceptibility profile of *Pasteurella multocida* strains originating from clinical cases in waterfowl in Hungary.

## 2. Materials and Methods

### 2.1. Isolation of the Bacterial Strains

All samples were isolated from clinical cases that occurred between February 2022 and May 2023, with the assistance of the National Food Chain Safety Office, Veterinary Diagnostic Directorate. The isolates were stored at −80 °C in a Microbank™ system (Pro-Lab Diagnostics, Richmond Hill, ON, Canada) until use. Information regarding the samples was recorded, including the species (duck, goose), the organ from which they were obtained (liver, lung, bone marrow), and the geographic origin. The species identification of the strains was determined using MALDI-TOF mass spectrometry (Flextra-LAB Kft., Budapest, Hungary) and the Biotyper software vs. 12.0 (Bruker Daltonics GmbH, Bremen, Germany, 2024) [21].

### 2.2. Antimicrobial Susceptibility Testing

The stock solutions of the tested antimicrobial agents (Merck KGaA, Darmstadt, Germany) were prepared according to the Clinical Laboratory Standards Institute (CLSI) guidelines [22] at a concentration of 1024 µg/mL. A total of 26 different antibiotics were administered and were based on the breakpoint values available in the CLSI guidelines for *Pasteurella multocida* derived from poultry, and resistance levels were determined for penicillin, amoxicillin–clavulanic acid, ceftiofur, spectinomycin, florfenicol, chloramphenicol, tilmicosin, and enrofloxacin.

The phenotypic expression of antimicrobial resistance was examined by determining the minimal inhibitory concentration (MIC) values of individual bacterial strains. This was conducted according to CLSI guidelines in cation-adjusted Mueller–Hinton broth (CAMHB) using 96-well microtiter plates (VWR International, LLC., Debrecen, Hungary). A two-fold dilution series was prepared, and bacterial suspension adjusted to a 0.5 McFarland standard was inoculated onto the plates [22]. Evaluation was performed using a Sensititre^TM^ SWIN^TM^ automatic MIC reader (ThermoFisher Scientific, Budapest, Hungary) and the VIZION system software vs. 3.4 (ThermoFisher Scientific, Budapest, Hungary, 2024). The breakpoints for each antimicrobial agent were determined based on the guidelines of CLSI [23]. The reference isolate used was *Escherichia coli* (ATCC 25922).

## 3. Results

### 3.1. Origin of the Isolates

**Figure 1:**
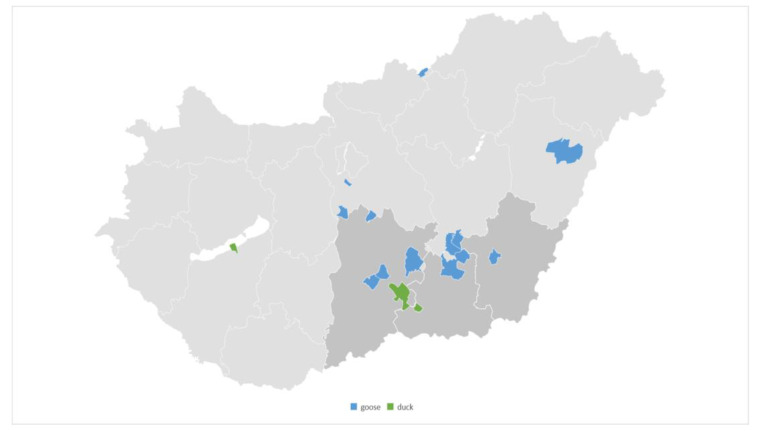
The distribution of the origin of our samples. Almost half of samples (48%) originated from the Dél-Alföld region (darker gray).

The origin of the 27 samples is illustrated in Figure 1, encompassing 15 different locations (Supplementary Table S1). The Hungarian waterfowl industry is concentrated in the Dél-Alföld region in the south of the country. Almost half of the samples (48%) originated from this region. The results of species identification using the MALDI-TOF device are presented in Supplementary Table S2. The results showed a perfect match for all *Pasteurella multocida* isolates, as we observed a logarithmic score bigger than two in all positive samples.

**Figure 2:**
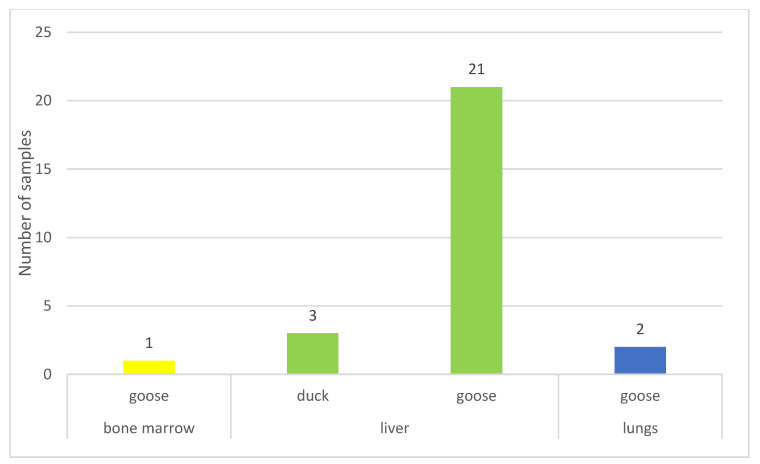
The organ-specific origin of the isolated strains that caused infection, categorized by species. Most infections affected geese, with the majority of bacteria isolated from the liver.

Figure 2 illustrates that the majority of samples (89%) originated from geese, with most samples (89%) having been isolated from the liver (Supplementary Table S1).

### 3.2. Antimicrobial Susceptibility Testing

During the determination of MIC values (Supplementary Table S3), all strains were susceptible to spectinomycin, penicillin, ceftiofur, tilmicosin, florfenicol, amoxicillin–clavulanic acid, and chloramphenicol. Regarding enrofloxacin, 44.4% of the strains exhibited intermediate susceptibility or resistance (Table 1). Taking into account the epidemiological cut-off (ECOFF) values, 7.4% of the strains were non-wild-type for ceftiofur, while 44.4% were non-wild-type for enrofloxacin. The representation of the ECOFF determined by the European Committee on Antimicrobial Susceptibility Testing (EUCAST), which separates wild-type from non-wild-type strains, is illustrated in the last column of Table 1. These values were higher than the calculated MIC_50_ and MIC_90_ values in all cases, except for the MIC_90_ of enrofloxacin.

The MIC_50_ and MIC_90_ values were obtained from the data for the following substances: penicillin (0.06 and 0.06), amoxicillin–clavulanic acid (0.125 and 0.125), ceftiofur (0.015 and 0.015), spectinomycin (8 and 8), florfenicol (0.25 and 0.25), chloramphenicol (0.5 and 0.5), tilmicosin (4 and 8), and enrofloxacin (0.015 and 1).

**Figure 3:**
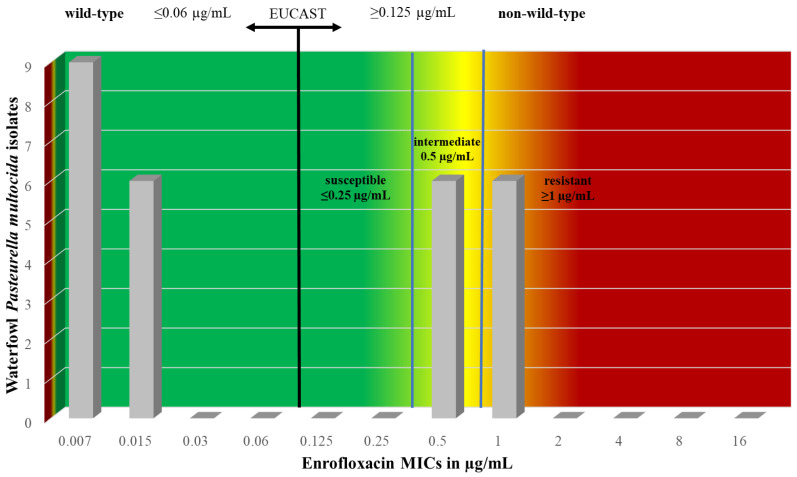
Comparison of the categories susceptible, intermediate, and resistant (clinical breakpoints) as well as wild-type and non-wild-type (EUCAST) using the combination enrofloxacin and *Pasteurella multocida*.

During the examination of antimicrobial agents with CLSI-derived breakpoint values, we observed six isolates with intermediate susceptibility and six isolates with resistance to enrofloxacin. The distribution of these isolates, as well as the representation of the ECOFF determined by EUCAST, which separates wild-type from non-wild-type strains, is illustrated in Figure 3.

Table 2 and Table 3 contain the MIC values of other antimicrobial agents that do not have breakpoint values, along with their MIC_50_ and MIC_90_ values. The exact MIC values for each strain are summarized in Supplementary Table S4.

In the absence of CLSI breakpoints, we assessed the sensitivity of the strains using EUCAST ECOFF values (Table 2). For tiamulin, the cut-off value is 64 µg/mL; the MIC_50_ and MIC_90_ values indicate that the tested population was sensitive. For potent sulphonamide, the cut-off value is 0.125 µg/mL, suggesting that the population was not sensitive. For amoxicillin, with an ECOFF value of 0.5 µg/mL, both MIC_50_ and MIC_90_ values confirm that the strains were susceptible. For doxycycline, the cut-off value is 1 µg/mL, indicating that 90% of the population tested was definitely sensitive. The cut-off values for cephalexin and gentamicin are 8 µg/mL, showing that at least 90% of the population was sensitive to both agents. For cefquinome, the threshold is 0.125 µg/mL, indicating that the tested strains were considered sensitive.

No ECOFF values have been established for cefotaxime, imipenem, tilozin, ceftriaxone, lincomycin, colistin, marbofloxacin, clindamycin, levofloxacin, gatifloxacin, and lincomycin–spectinomycin. However, based on the MIC_50_ and MIC_90_ values, the strains are presumed to be susceptible to cefotaxime, imipenem, ceftriaxone, levofloxacin, and gatifloxacin (Table 3).

## 4. Discussion

During our research between 2022 and 2023, we conducted antimicrobial susceptibility testing of 27 clinical cases of isolated *Pasteurella multocida* strains using a total of 26 different antimicrobial agents. We determined the MIC values of the strains using the microdilution method, and in eight cases, we found that derived breakpoint values determined the resistance level. This zoonotic pathogen poses a risk to human health as well, making its role in public health significant. Opportunistic infections originating from surface wounds are quite prevalent, particularly among older adults and individuals with compromised immune systems [24]. Additionally, clinical cases of urinary tract infections and bacteremic meningitis have been reported. However, deaths caused by *Pasteurella* infections are rare, with the number of human deaths attributable to this infection ranging from 2 to 25 annually in the USA between 1993 and 2006 [13]. The disease continues to be present in semi-confined waterfowl husbandry systems, where outdoor access to the environment and water is provided, often affecting both domestic and wild waterfowl. Infection in these settings can lead to significant economic losses if a flock becomes infected [24].

The literature on the Hungarian situation is relatively scarce, and the examination results are based on the disk diffusion method [25]. Sellyei et al., examining strains isolated between 2005 and 2008, did not detect resistance to florfenicol, penicillin, and chloramphenicol, similar to our findings. Furthermore, they did not report resistance to enrofloxacin [26]. In contrast, we observed that 22% of the strains were resistant to enrofloxacin and that 22% had intermediate susceptibility to this antibiotic. In the strains isolated by Sellyei et al. between 2005 and 2010, 2.3% were resistant to penicillins (penicillin, amoxicillin–clavulanic acid) and 61.5% were resistant to fluoroquinolones (nalidixic acid, flumequine, enrofloxacin). Additionally, in 80% of cases, multidrug resistance [27] was observed. In contrast, we observed resistance only to enrofloxacin (22%) and a decrease in susceptibility to enrofloxacin (22%). This corresponds to the decreasing trend observed in phenotypic resistance over time. Thus, most agents have retained their effectiveness, and the proportion of enrofloxacin-resistant and multidrug-resistant strains is showing a decreasing trend. This could be explained by a reduction in antibiotic usage caused by two factors: firstly, the development of infrastructure, which has contributed to better hygiene and has thus reduced predisposing factors and led to the reduced use of antibiotics, and secondly, the tightening of and adherence to international guidelines for reducing antibiotic use.

Compared to the international situation, a similar study was conducted using the microdilution method in Korea in 2021 [28]. In accordance with our results, no resistant strains were found for amoxicillin–clavulanic acid, ceftiofur, spectinomycin, and tilmicosin in either Korea or Hungary. However, 11.1% of the strains were resistant to florfenicol in the Korean study. Similar resistance was not detected in our study examining the Hungarian strains. Conversely, enrofloxacin resistance was not found in Korea, whereas 20% of the Hungarian samples showed resistance to enrofloxacin and 20% showed intermediate susceptibility. Enrofloxacin is an approved fluoroquinolone antibiotic for poultry. Its mode of action is concentration-dependent bactericidal activity, achieved by inhibiting bacterial DNA replication and transcription. It is particularly effective in treating infections caused by *Pasteurella* species. However, during therapeutic use, it is essential to ensure that the ratio of the total amount of the compound in the body to the MIC over a 24 h treatment period (AUC_0–24_) is at least 125. When considering the ratio of the maximum drug concentration (C_max_) to MIC in tissues, it should be around 8–10 [29,30,31]. The rapid development of resistance to enrofloxacin necessitates a significant reduction in its future use [32] and a partial or complete replacement of antibiotics with promising agents such as antimicrobial peptides [33], propolis [34,35], or plant essential oils [36]. The observed decrease in susceptibility and the emergence of resistance raises concerns, warranting further investigation of strains through metagenomic analysis facilitated by next-generation sequencing.

In this study, we determined the antimicrobial susceptibility patterns of *Pasteurella multocida* strains isolated from waterfowl in Hungary. Since the antibiotic sensitivity profile of a bacterium is important for its general characterization, we also tested some antibiotics that are not typically used for treating diseases caused by *Pasteurella multocida*. The MIC_50_ and MIC_90_ values were ≤0.12 µg/mL and ≤0.12 µg/mL for ceftiofur, 8 µg/mL and 16 µg/mL for spectinomycin, and 0.5 µg/mL and ≥8 µg/mL for florfenicol [28]. In our study, we obtained values of 0.015 µg/mL and 0.015 µg/mL for ceftiofur, 8 µg/mL and 8 µg/mL for spectinomycin, and 0.25 µg/mL and 0.25 µg/mL for florfenicol. The higher MIC_90_ values observed for enrofloxacin (compared to the EUCAST ECOFF value) suggest the presence of antimicrobial resistance genes in intermediate-resistant and resistant strains. Many international studies, however, also use the disk diffusion method, so we had little basis for comparison with the results of our test method. Using this method, Eid et al. observed that 87.5% of the strains were resistant to spectinomycin and penicillin and that 12.5% were resistant to chloramphenicol [37]. Kamruzzaman et al. reported that 100% of the strains were resistant to penicillin [38]. Furian et al. found that 1.8% of the strains were resistant to amoxicillin, 1.8% were resistant to ceftiofur, and 23.2% were resistant to enrofloxacin [39]. Shivachandra et al. detected resistance rates of 26% resistance for chloramphenicol and 28.5% for enrofloxacin [40]. Xiao et al. reported that 66.7% of the strains were resistant to spectinomycin, 100% were resistant to chloramphenicol, and 27.8% were resistant to amoxicillin [41]. However, Tan et al. did not find resistance to amoxicillin, florfenicol, enrofloxacin, and spectinomycin [42]. Eid et al. reported resistance rates of 30% for amoxicillin and 60% for neomycin. Additionally, resistance rates of 30% for potent sulfonamide [37] and 56.9% for doxycycline [40] were observed. Amoxicillin is one of the primary choices for treating *Pasteurella multocida* infections [43], with ECOFF values suggesting that the strains under investigation would be susceptible. The third- and fourth-generation cephalosporins (cefotaxime, ceftriaxone, cephalexin, cefquinome) and imipenem all exhibited very low MIC values, which is particularly significant from a public health perspective, as these agents are used for life-threatening infections requiring intravenous administration in hospital settings [44]. *Pasteurella multocida* is reported to be resistant to lincosamides (lincomycin, clindamycin) while showing moderate sensitivity to pleuromutilins (tiamulin) and macrolides (tilozin), as confirmed by the high MIC values for these agents [45]. Although aminoglycosides like gentamicin may be sensitive in vitro, they are not absorbed orally, making them impractical for poultry treatment due to the impracticality of parenteral administration [46,47]. Similarly, colistin is not absorbed when administered orally [48].

In many cases, our results coincide with previous results in both domestic and international studies. However, it should be emphasized that most studies use the disk diffusion method, in contrast to the microdilution method we applied. Microdilution is considered the gold standard, and in the future, it is advisable to conduct more regular surveys using microdilution in the waterfowl industry, especially concerning *Pasteurella multocida*. The active substances (penicillin, amoxicillin) commonly used for treating *Pasteurella multocida* infections in poultry demonstrated excellent sensitivity. Furthermore, the third- and fourth-generation cephalosporins or fluoroquinolones, which are critically important active substances for public health, also retained their efficacy.

## 5. Conclusions

Overall, it can be concluded that *Pasteurella multocida* retained its susceptibility to most of the tested antimicrobial agents. The appearance of resistance to enrofloxacin, a fluoroquinolone, supports the notion of overuse in the poultry industry, which is particularly concerning considering the significant public health implications of this drug class. Fluoroquinolones are critically important active substances reserved for hospital inpatient care. Therefore, efforts to curb its use are essential to preserve its efficacy in human health care. Furthermore, there is a need for pharmacokinetic and pharmacodynamic in vivo studies specific to poultry species to establish species-specific breakpoint values. This would enable an exact evaluation of the in vitro results and would more precisely guide antibiotic treatment for commercial livestock.

## Figures and Tables

**Table 1:** The distribution of MIC values for *Pasteurella multocida* isolates against the tested antimicrobial agents, as well as MIC_50_, MIC_90_, and epidemiological cut-off value (ECOFF) values. For each antimicrobial agent, the upper rows indicate the number of isolates, while the lower rows show the percentage distribution. The red vertical lines represent the resistance breakpoint, and the green vertical lines indicate the breakpoint for intermediate susceptibility according to Clinical Laboratory Standards Institute (CLSI) recommendations.

ECOFF (µg/mL) ^4^	MIC_90_ (µg/mL) ^3^	MIC_50_ (µg/mL) ^2^	0.007	0.015	0.03	0.06	0.125	0.25	0.5	1	2	4	8	16	Breakpoint ^1^ (µg/mL)	Antibiotic
0.5	0.06	0.06				24	3								≥1	Penicillin
						88.9	11.1									
0.25	0.125	0.125				2	25								≥1	Amoxicillin–clavulanic acid ^5^
						7.4	92.6									
0.06	0.015	0.015		25		2									≥8	Ceftiofur
				92.6		7.4										
64	8	8										2	24	1	≥128	Spectinomycin
												7.4	88.9	3.7		
1	0.25	0.25						27							≥8	Florfenicol
								100								
1	0.5	0.5						1	26						≥32	Chloramphenicol
								3.7	96.3							
32	8	4									1	14	10	2	≥32	Tilmicosin
											3.7	51.9	37	7.4		
0.06	1	0.015	9	6					6	6					≥1	Enrofloxacin
			33.3	22.2					22.2	22.2						

^1^ CLSI; ^2^ MIC_50_: antimicrobial concentration at which 50% of the isolates were inhibited; ^3^ MIC_90_: antimicrobial concentration at which 90% of the isolates were inhibited; ^4^ EUCAST; ^5^ 4:1 ratio.

**Table 2:** The distribution of MIC values for *Pasteurella multocida* isolates against the tested antimicrobial agents, as well as MIC_50_, MIC_90_, and epidemiological cut-off value (ECOFF) values. For each antimicrobial agent, the upper rows indicate the number of isolates, while the lower rows show the percentage distribution. For the substances listed in the table, no CLSI breakpoints are available; only EUCAST ECOFF values are provided.

ECOFF (µg/mL) ^3^	MIC_90_ (µg/mL) ^2^	MIC_50_ (µg/mL) ^1^	0.007	0.015	0.03	0.06	0.125	0.25	0.5	1	2	4	8	16	32	64	128	Antibiotic
64	32	16											3	16	5	2	1	Tiamulin
													11.1	59.3	18.5	7.4	3.7	
0.125	8	1				1	2		5	10		1	5		3			Potent sulphonamide ^4^
						3.7	7.4		18.5	37.0		3.7	18.5		11.1			
0.5	0.125	0.125					27											Amoxicillin
							100.0											
1	0.125	0.03		10	8	5	1				1			2				Doxycycline
				37.0	29.6	18.5	3.7				3.7			7.4				
8	4	2									17	9		1				Cephalexin
											63.0	33.3		3.7				
8	2	2									25	2						Gentamicin
											92.6	7.4						
0.125	0.03	0.03			27													Cefquinome
					100.0													

^1^ MIC_50_: antimicrobial concentration at which 50% of the isolates were inhibited; ^2^ MIC_90_: antimicrobial concentration at which 90% of the isolates were inhibited; ^3^ EUCAST; ^4^ trimethoprim–sulphamethoxazole (1:19 ratio).

**Table 3:** The distribution of MIC values for *Pasteurella multocida* isolates against the tested antimicrobial agents, as well as MIC_50_, MIC_90_, and epidemiological cut-off value (ECOFF) values. For each antimicrobial agent, the upper rows indicate the number of isolates, while the lower rows show the percentage distribution. The table displays the scopes for which neither breakpoints nor ECOFF values are available.

MIC_90_ (µg/mL) ^2^	MIC_50_ (µg/mL) ^1^	0.007	0.015	0.03	0.06	0.125	0.25	0.5	1	2	4	8	16	32	64	128	Antibiotic
0.015	0.015		27														Cefotaxime
			100														
0.25	0.25				3	7	16	1									Imipenem
					11.1	25.9	59.3	3.7									
32	32												3	24			Tilozin
													11.1	88.9			
0.015	0.015	1	25	1													Ceftriaxone
		3.7	92.6	3.7													
32	32												1	26			Lincomycin
													3.7	96.3			
4	2						2	3	7	7	6	1	1				Colistin
							7.4	11.1	25.9	25.9	22.2	3.7	3.7				
2	0.06		3	7	4	1			6	6							Marbofloxacin
			11.1	25.9	14.8	3.7			22.2	22.2							
128	64												1	6	15	5	Clindamycin
													3.7	22.2	55.6	18.5	
0.5	0.015	5	8	1			2	11									Levofloxacin
		7.4	29.6	3.7			7.4	40.7									
0.5	0.06	2	8	1	1	10	4										Gatifloxacin
		7.4	29.6	3.7	3.7	37	14.8										
32	32												2	25			Lincomycin–spectinomycin ^3^
													7.4	92.6			

^1^ MIC_50_: antimicrobial concentration at which 50% of the isolates were inhibited; ^2^ MIC_90_: antimicrobial concentration at which 90% of the isolates were inhibited; ^3^ lincomycin–spectinomycin (2:1 ratio).

## Data Availability

The data presented in this study are available from the corresponding author upon reasonable request.

## Supplementary Materials

The following supporting information can be downloaded at https://www.mdpi.com/article/10.3390/vetsci11050194/s1, Table S1: Origin of *Pasteurella multocida* strains isolated from clinical cases by animal species, organs, and regional and local origins in Hungary; Table S2: The results of species identification using the MALDI-TOF device; Table S3: MIC values per strain of active substances with CLSI-derived breakpoints for *Pasteurella multocida*; Table S4: MIC values for active substances without clinical CLSI breakpoints for *Pasteurella multocida* strains and their MIC_50_ and MIC_90_ values.
