# Supplementary material for: Antimicrobial Susceptibility Profiles of Pasteurella multocida Isolates from Clinical Cases of Waterfowl in Hungary between 2022 and 2023
Source: Vet Sci. 2024 Apr 28;11(5):194. doi: 10.3390/vetsci11050194 (PMC11125817; doi:10.3390/vetsci11050194)
Supplement: Supplementary file 1 [file vetsci-11-00194-s001.zip › vetsci-2953424-supplementary.pdf]

Supplementary Table S1: Origin of Pasteurella multocida strains isolated from clinical cases by animal species, organs, and regional and local origins in Hungary.

No.	Bacteria	ID	Species	Organ	Regio	Settlement
1	*Pasteurella multocida*	10906	goose	liver	Észak-Alföld	Kunszentmárton
2	*Pasteurella multocida*	11298	goose	liver	Dél-Alföld	Kiskunfélegyháza
3	*Pasteurella multocida*	11298	goose	lungs	Dél-Alföld	Kiskunfélegyháza
4	*Pasteurella multocida*	11788	goose	liver	Dél-Alföld	Kondoros
5	*Pasteurella multocida*	14832	goose	liver	Közép-Magyarország	Tatárszentgyörgy
6	*Pasteurella multocida*	15704	goose	liver	Észak-Magyarország	Cered
7	*Pasteurella multocida*	21958	goose	liver	Dél-Alföld	Szentes
8	*Pasteurella multocida*	21958	goose	lungs	Dél-Alföld	Szentes
9	*Pasteurella multocida*	22079	duck	liver	Dél-Dunántúl	Balatonszemes
10	*Pasteurella multocida*	22919	goose	liver	Dél-Alföld	Szentes
11	*Pasteurella multocida*	25054	goose	liver	Dél-Alföld	Szentes
12	*Pasteurella multocida*	37287	goose	liver	Dél-Alföld	Soltvadkert
13	*Pasteurella multocida*	41550	goose	liver	Közép-Magyarország	Dömösd
14	*Pasteurella multocida*	52681	goose	liver	Dél-Alföld	Bócsa
15	*Pasteurella multocida*	52682	goose	liver	Dél-Alföld	Bócsa
16	*Pasteurella multocida*	57991	goose	liver	Dél-Alföld	Bócsa
17	*Pasteurella multocida*	58612	goose	liver	Észak-Alföld	Öcsöd
18	*Pasteurella multocida*	60458	goose	liver	Észak-Alföld	Öcsöd
19	*Pasteurella multocida*	60463	goose	liver	Észak-Alföld	Öcsöd
20	*Pasteurella multocida*	66107	goose	liver	Észak-Alföld	Öcsöd
21	*Pasteurella multocida*	71764	goose	bone marrow	Észak-Alföld	Öcsöd
22	*Pasteurella multocida*	72106	goose	liver	Észak-Alföld	Öcsöd
23	*Pasteurella multocida*	81753	goose	liver	Közép-Magyarország	Taksony
24	*Pasteurella multocida*	90742	duck	liver	Dél-Alföld	Kiskunmajsa
25	*Pasteurella multocida*	92197	duck	liver	Dél-Alföld	Forráskút
26	*Pasteurella multocida*	96174	goose	liver	Közép-Magyarország	Tatárszentgyörgy
27	*Pasteurella multocida*	106347	goose	liver	Észak-Alföld	Debrecen

Supplementary Table S2: The results of species identification using the MALDI-TOF device.

No.	Matched Pattern	log (score) (Conf.)	*NCBI Identifier
1	*Pasteurella multocida* ssp *multocida* CIP 56_3 CIP	2.44 (+++)	747
2	*Pasteurella multocida* A244_07 FLR	2.27 (+++)	747
3	*Pasteurella multocida* A244_07 FLR	2.20 (+++)	747
4	*Pasteurella multocida* A244_07 FLR	2.27 (+++)	747
5	*Pasteurella multocida* besSt7 THL	2.21 (+++)	747
6	*Pasteurella multocida* A244_07 FLR	2.46 (+++)	747
7	*Pasteurella multocida* A244_07 FLR	2.26 (+++)	747
8	*Pasteurella multocida* ssp *multocida* CIP 56_3 CIP	2.33 (+++)	747
9	*Pasteurella multocida* A244_07 FLR	2.31 (+++)	747
10	*Pasteurella multocida* A244_07 FLR	2.31 (+++)	747
11	*Pasteurella multocida* A244_07 FLR	2.37 (+++)	747
12	*Pasteurella multocida* A129_06 FLR	2.29 (+++)	747
13	*Pasteurella multocida* A244_07 FLR	2.34 (+++)	747
14	*Pasteurella multocida* A129_06 FLR	2.21 (+++)	747
15	*Pasteurella multocida* A244_07 FLR	2.34 (+++)	747
16	*Pasteurella multocida* ssp *multocida* CIP 56_3 CIP	2.27 (+++)	747
17	*Pasteurella multocida* A244_07 FLR	2.38 (+++)	747
18	*Pasteurella multocida* A244_07 FLR	2.26 (+++)	747
19	*Pasteurella multocida* ssp *multocida* CIP 56_3 CIP	2.27 (+++)	747
20	*Pasteurella multocida* ssp *multocida* CIP 56_3 CIP	2.24 (+++)	747
21	*Pasteurella multocida* ssp *multocida* CIP 56_3 CIP	2.28 (+++)	747
22	*Pasteurella multocida* ssp *multocida* CIP 56_3 CIP	2.37 (+++)	747
23	*Pasteurella multocida* ssp *multocida* CIP 56_3 CIP	2.30 (+++)	747
24	*Pasteurella multocida* A244_07 FLR	2.19 (+++)	747
25	*Pasteurella multocida* ssp *multocida* CIP 56_3 CIP	2.27 (+++)	747
26	*Pasteurella multocida* A244_07 FLR	2.24 (+++)	747
27	*Pasteurella multocida* A244_07 FLR	2.34 (+++)	747

+++ High Confidence Identification (2.00 – 3.00 range)

*NCBI: National Center for Biotechnology Information

Supplementary Table S3: MIC values per strain of active substances with CLSI-derived breakpoints for Pasteurella multocida.

**No.**	**PEN**	**AMC**	**CTO**	**SPE**	**FLO**	**CLO**	**TIM**	**ENR**
	**MIC (µg/mL)**							
1	0.06	0.125	0.015	8	0.25	0.5	4	1
2	0.06	0.125	0.015	8	0.25	0.5	8	1
3	0.125	0.125	0.015	4	0.25	0.5	4	0.5
4	0.06	0.125	0.015	8	0.25	0.5	2	0.015
5	0.06	0.125	0.015	8	0.25	0.5	4	0.015
6	0.06	0.125	0.06	8	0.25	0.5	4	0.007
7	0.125	0.125	0.015	4	0.25	0.5	4	0.007
8	0.06	0.125	0.015	8	0.25	0.5	4	0.007
9	0.06	0.125	0.015	8	0.25	0.5	8	0.015
10	0.06	0.125	0.015	8	0.25	0.5	16	0.015
11	0.06	0.125	0.015	8	0.25	0.5	8	0.007
12	0.06	0.125	0.015	8	0.25	0.5	4	0.5
13	0.06	0.125	0.06	8	0.25	0.25	4	0.015
14	0.125	0.125	0.015	8	0.25	0.5	8	1
15	0.06	0.125	0.015	8	0.25	0.5	4	1
16	0.06	0.06	0.015	8	0.25	0.5	8	0.007
17	0.06	0.125	0.015	8	0.25	0.5	16	0.007
18	0.06	0.125	0.015	8	0.25	0.5	8	0.5
19	0.06	0.06	0.015	8	0.25	0.5	8	0.5
20	0.06	0.125	0.015	8	0.25	0.5	4	0.5
21	0.06	0.125	0.015	8	0.25	0.5	4	0.5
22	0.06	0.125	0.015	8	0.25	0.5	8	1
23	0.06	0.125	0.015	8	0.25	0.5	4	0.007
24	0.06	0.125	0.015	8	0.25	0.5	8	0.015
25	0.06	0.125	0.015	8	0.25	0.5	8	1
26	0.06	0.125	0.015	16	0.25	0.5	4	0.015
27	0.06	0.125	0.015	8	0.25	0.5	4	0.007

PEN - penicillin; AMC - amoxicillin-clavulanic acid; CTO - ceftiofur; SPE - spectinomycin; FLO - florfenicol; CLO - chloramphenicol; TIM - tilmicosin; ENR – enrofloxacin

Supplementary Table S4: MIC values for active substances without clinical CLSI breakpoints for Pasteurella multocida strains and their MIC_{50} and MIC_{90} values.

**No.**	**TIA**	**PSA**	**CXM**	**IMI**	**TIL**	**CTR**	**AMX**	**LIN**	**COL**	**DOX**	**MAR**	**CLX**	**GEN**	**CLI**	**LEV**	**CFQ**	**GAT**	**LSP**
	**MIC (µg/mL)**																	
1	16	0.5	0.015	0.25	32	0.015	0.125	32	2	0.015	2	4	2	64	0.5	0.03	0.5	16
2	32	0.5	0.015	0.25	32	0.015	0.125	32	16	0.015	2	2	2	64	0.5	0.03	0.5	32
3	16	1	0.015	0.25	32	0.015	0.125	32	2	0.03	1	4	2	64	0.25	0.03	0.25	32
4	32	32	0.015	0.25	32	0.015	0.125	32	0.5	0.06	0.03	4	2	128	0.007	0.03	0.03	32
5	8	8	0.015	0.125	16	0.015	0.125	32	0.25	0.06	0.06	2	2	32	0.015	0.03	0.015	32
6	8	8	0.015	0.5	32	0.03	0.125	32	4	0.06	0.03	2	2	64	0.007	0.03	0.015	32
7	16	0.125	0.015	0.25	32	0.015	0.125	32	2	0.015	0.015	4	2	64	0.5	0.03	0.25	32
8	16	4	0.015	0.06	32	0.007	0.125	16	4	0.015	0.015	2	2	32	0.007	0.03	0.06	32
9	32	32	0.015	0.25	32	0.015	0.125	32	4	2	0.06	4	2	64	0.015	0.03	0.015	32
10	32	8	0.015	0.125	32	0.015	0.125	32	2	0.125	0.015	4	2	128	0.007	0.03	0.015	32
11	128	32	0.015	0.25	32	0.015	0.125	32	1	16	0.03	16	2	128	0.015	0.03	0.015	32
12	16	0.06	0.015	0.25	32	0.015	0.125	32	0.5	0.03	1	2	2	32	0.5	0.03	0.25	32
13	16	1	0.015	0.25	16	0.015	0.125	32	0.5	0.015	0.125	4	2	32	0.03	0.03	0.03	32
14	32	1	0.015	0.25	32	0.015	0.125	32	1	0.06	2	2	2	64	0.5	0.03	0.25	32
15	16	8	0.015	0.25	32	0.015	0.125	32	2	0.03	2	2	2	64	0.5	0.03	0.5	32
16	64	1	0.015	0.06	32	0.015	0.125	32	2	16	0.03	4	4	128	0.015	0.03	0.015	32
17	64	1	0.015	0.06	32	0.015	0.125	32	1	0.06	0.06	4	2	128	0.015	0.03	0.015	32
18	16	1	0.015	0.125	32	0.015	0.125	32	1	0.015	1	2	2	64	0.5	0.03	0.25	32
19	16	0.5	0.015	0.125	32	0.015	0.125	32	1	0.03	2	2	2	32	0.5	0.03	0.25	32
20	16	0.5	0.015	0.125	32	0.015	0.125	32	1	0.015	2	2	2	64	0.5	0.03	0.25	32
21	16	1	0.015	0.25	32	0.015	0.125	32	4	0.015	1	2	2	64	0.5	0.03	0.25	32
22	16	1	0.015	0.25	32	0.015	0.125	32	4	0.015	1	2	2	64	0.25	0.03	0.25	32
23	16	0.125	0.015	0.125	32	0.015	0.125	32	2	0.03	0.03	2	2	64	0.007	0.03	0.007	32
24	16	8	0.015	0.25	16	0.015	0.125	32	1	0.015	0.06	2	2	32	0.015	0.03	0.25	32
25	16	1	0.015	0.25	32	0.015	0.125	32	8	0.03	1	2	2	16	0.5	0.03	0.5	32
26	8	0.5	0.015	0.25	32	0.015	0.125	32	0.25	0.03	0.03	2	2	64	0.015	0.03	0.015	16
27	16	1	0.015	0.125	32	0.015	0.125	32	4	0.03	0.03	2	4	64	0.015	0.03	0.007	32
**MIC_{50}**	16	1	0.015	0.25	32	0.015	0.125	32	2	0.03	0.06	2	2	64	0.015	0.03	0.06	32
**MIC_{90}**	32	8	0.015	0.25	32	0.015	0.125	32	4	0.125	2	4	2	128	0.5	0.03	0.5	32

TIA - tiamulin; PSA - potent sulphonamide (trimethoprim - sulphamethoxazole, ratio 1:19); CXM - cefotaxime; IMI - imipenem; TIL - tilozin; CTR - ceftriaxone; AMX - amoxicillin; LIN - lincomycin; COL - colistin; DOX - doxycycline; MAR - marbofloxacin; CLX - cefalexin; GEN - gentamicin; CLI - clindamycin; LEV - levofloxacin; CFQ - cefquinome; GAT - gatifloxacin; LSP – lincomycin - spectinomycin (ratio 2:1)
